# Supplementary material for: Estimation of the Minimum Effective Dose of Dietary Supplement Crocetin for Prevention of Myopia Progression in Mice
Source: Nutrients. 2020 Jan 9;12(1):180. doi: 10.3390/nu12010180 (PMC7019851; doi:10.3390/nu12010180)
Supplement: Supplementary file 1 [file nutrients-12-00180-s001.pdf]

Supplementary Information for

Estimation of the minimum effective dose of dietary supplement crocetin for prevention of myopia progression in mice

Kiwako Mori[1,2], Toshihide Kurihara[1,2,*], Xiaoyan Jiang[1,2], Shin-ichi Ikeda[1,2], Erisa Yotsukura[1,2], Hidemasa Torii[1,2], Kazuo Tsubota[1,*]

Table of Contents:

Table S1. Liquid chromatography-mass spectrometry (LC-MS) analysis condition

S1 Table. Liquid chromatography-mass spectrometry (LC-MS) analysis condition

LC condition	
HPLC	ACQUITY UPLC I-Class Systems (Waters)
Analytical column	InertSustain C18 (5 μm, 2.1 mm I.D. × 50 mm, GL Sciences)
Mobile phase	A: Experimental water/1 mol/L ammonium acetate (100:1, v/v)
	B: Methanol
Flow rate	0.5 mL/min
Gradient table	

Time（min）	A（%）	B（%）
Initial	70	30
1.50	1	99
2.50	1	99
3.00	70	30

Column temperature	40°C
Sample cooler	10°C
Injection volume	30 μL
Injector washing solution	Acetonitrile/2-propanol/ methanol/dimethyl sulfoxide/experimental water (1:1:1:1:1, v/v/v/v/v)
MS/MS condition	

MS	Xevo TQ-XS (Waters)
Scan type	MRM (Multiple Reaction Monitoring)
Ionization mode	ESI (Electrospray ionization)
Polarity	Negative
Cone (V)	30
Capillary (kV)	1
Monitor ion and Collision energy (CE)	

Analyte	Monitor ion	CE (eV)
Crocetin	m/z = 327.2 → 239.0	10
I.S. (Niflumic acid)	m/z = 281.0 → 237.0	16
